# Psychometric Properties of the Athens Insomnia Scale in Occupational Computer Users

**DOI:** 10.3390/healthcare8020089

**Published:** 2020-04-07

**Authors:** Mohamed Sherif Sirajudeen, Md. Dilshad Manzar, Mazen Alqahtani, Msaad Alzhrani, Abdulrhman Albougami, Padmakumar Somasekharan Pillai, D. Warren Spence, Seithikurippu R. Pandi-Perumal

**Affiliations:** 1 Department of Physical Therapy and Health Rehabilitation, College of Applied Medical Sciences, Majmaah University, Majmaah 11952, Saudi Arabia; m.sirajudeen@mu.edu.sa (M.S.S.); mm.alqahtani@mu.edu.sa (M.A.); m.alzhrani@mu.edu.sa (M.A.); 2 Department of Nursing, College of Applied Medical Sciences, Majmaah University, Majmaah 11952, Saudi Arabia; a.albougami@mu.edu.sa; 3 Yenepoya Physiotherapy College, Yenepoya (Deemed to be University), Mangalore 575018, Karnataka, India; padhupt@gmail.com; 4 Independent researcher, 652 Dufferin Street, Toronto, ON M6K 2B4, Canada; dwspence@fastmail.fm; 5 Somnogen Canada Inc., College Street, Toronto, ON M6K 2B4, Canada; pandiperumal2020@gmail.com

**Keywords:** insomnia, validity, dimensionality, consistency, reliability, Indian

## Abstract

*Background:* Various studies have shown that insomnia is associated with computer use. The Athens Insomnia Scale (AIS) is an 8-item tool that has been widely used for screening insomnia. No studies have investigated the psychometric validity of AIS in occupational computer users. *Objective:* the current research aimed to test the psychometric properties of the AIS among occupational computer users. Materials and Methods: a sample of four hundred and twenty-four occupational computer users (age: 20–65 years and body mass index: 21.6 ± 3.5 kg/m^2^) completed an AIS and a socio-demographic questionnaire in this cross-sectional study. *Results:* a confirmatory factor analysis demonstrated that the three-factor model had an adequate fit (the goodness of fit index (0.95), incremental fit index (0.90) and χ^2^/df (2.61)). Evidence was found for configural, scalar and metric invariance of the 3-factor model across gender groups. A moderate level of internal consistency was implied by a Cronbach’s alpha of 0.66. *Conclusion:* the findings of the present research support the validity of AIS for screening insomnia, as demonstrated by the scale’s psychometric properties; its internal consistency, internal homogeneity, item discrimination, and factorial validity.

## 1. Introduction

According to the most recent manual by the American Association of Sleep Medicine, insomnia is defined by the presence of constant sleep problems, which may be actual or perceived, together with disturbed socio-occupational functioning during the day, despite having had an adequate opportunity for sleep [1]. The prevalence of insomnia is continuously increasing across many countries of the world [2,3,4,5,6], an issue which is particularly important from a public health standpoint, inasmuch as insomnia, which is at the root of several other health complications, is often trivialized by sufferers as being a lifestyle problem rather than a significant health concern, and, finally, because its diagnostic importance continues to be de-emphasized or even ignored in most routine medical examinations. As a consequence, efforts to treat the condition are minimal [2,7]. It is particularly relevant to these considerations that some of the causes of insomnia may be an under-recognized consequence of modern technology, and as such may be preventable. A growing number of studies are showing for instance that sleep disruption or insomnia may be the result of computer use in the workplace. Insomnia is associated with computer use after adjustment for multiple confounders [8]. 

Insomnia was associated with extended visual display terminal (VDT) work, among Japanese government employees [9]. Even minimum or moderate levels of computer users are significant predictors of insomnia among Greek adolescents [10]. Daily VDT work was associated with many aspects of insomnia-related disturbances, including delays in sleep onset, sleep interruptions (wake after sleep onset), waking before the desired waking time, poor sleep quality, reduced sleep duration, daytime sleepiness, and psychosocial dysfunctions during the day [11]. 

An increasing amount of evidence indicates that a bidirectional relationship exists between psychosomatic conditions and insomnia and that this relationship may be exacerbated by excessive computer use [12,13,14]. The psychological and physical effects of insomnia have secondary consequences for the social and employment stability of affected individuals. These may result in disrupted social relationships, marital instability, and workplace accidents, all of which may add to the economic burden that must be borne by society generally [15].

The socio-economic impact of insomnia underscores the need for continued and concerted long-term research efforts into its causes and remediation. It is evident that whatever therapeutic strategies are developed for treating workplace-related insomnia, whether this is due to computer use or other factors, it is clear that the identification of its presence would be an essential first step. It is also clear that an easily administered psychometric screening tool would represent an ideal means for screening insomnia. This would provide critical information regarding the severity of the problem in any affected individuals and, further, would alert management to how widespread the problem was among their employees who use computers. 

The Athens Insomnia Scale (AIS) is an eight-item tool which is used to quantify the presence of insomnia. This instrument is widely used in clinical practice and research settings in the general population as well as in occupational medicine. The questionnaire is based on the ICD—ten criteria which are used to diagnose insomnia [16,17]. Up to the present time, no research has been conducted to assess the validity of the AIS in Indians or computer users in general. Given these considerations, the current study investigated the psychometric validity of the AIS in occupational computer users.

## 2. Material and Methods

### 2.1. Participants and Study Design

Participants in this cross-sectional study were recruited from among employees of educational institutions, hospitals and software companies in Karnataka, India. The primary inclusion criterion was that the employee must have a job that required the use of a desktop computer for a minimum of 3 h per day or 20 h per week [18,19]. Four hundred and twenty-four subjects between the ages of 20–65 years and a body mass index of 21.6 ± 3.5 kg/m^2^ completed this study. The Yenepoya (Deemed to be University) Ethics Committee, Yenepoya (Deemed to be University), India, reviewed and approved the research plan and its administration. The guidelines of Good Clinical Practice and the norms of the 2002 Declaration of Helsinki (DoH) were followed. 

### 2.2. Procedures and Measurements

All the participants were given detailed information about the aims and procedures of the study. Following that, a signed informed consent form was obtained from participants. An interviewer-administered study questionnaire package in English, including the Athens Insomnia Scale (AIS) and semi-structured socio-demographic questionnaire, was administered to all participants. 

### 2.3. Athens Insomnia Scale

Out of the eight AIS items, the first five address the participant’s nighttime symptoms (difficulty in sleep initiation, difficulty in maintaining sleep and early morning awakening), while the last three items address the daytime impact due to any reported sleep disturbances. This impact includes the participant’s subjective evaluation of his sense of wellbeing, functioning capacity and daytime sleepiness. Each item of the AIS is rated on an ordinal scale of 0–3, (with 0 corresponding to “no problem at all” and 3 to a “very serious problem”). The respondents were required to provide a positive rating if they had experienced a sleep difficulty described in each item at least three times a week during the last month. A maximum total score of 24, indicating the most severe symptoms of insomnia, was possible, while a cut-off point of ≥6 represented a minimum criterion for the confirmation of insomnia symptoms [16]. 

### 2.4. Socio-Demographic Questionnaire

A semi-structured socio-demographic questionnaire was used. This consisted of seven items, of which three were open-ended and four were close-ended. These items provide information regarding the respondent’s age, gender, height, weight, marital status, educational level, and smoking habits. 

## 3. Statistical Analysis

The statistical analysis was performed using SPSS version 23.0 SPSS (SPSS Inc., Chicago, IL, USA) and Factor 10.8.04 (Rovira i Virgili University, Tarragona, Spain). Mean with standard deviation, frequency, and the percentage was employed for the descriptive presentation of the participants’ characteristics. Item score distribution was shown using skewness, kurtosis, and frequency of respondents for each possible score. Three measures, i.e., Cronbach’s alpha if item deleted, Spearman’s item-total correlation coefficient and corrected item-total correlation coefficient were used as indicators of item discrimination as well as internal homogeneity.

The suitability of AIS scores for factor analysis was assessed using the Kaiser–Meyer–Olkin test, communality, Bartlett’s test of sphericity, determinant and estimating the diagonal element of the anti-image correlation matrix. The study sample was split into two equal sub-samples randomly to perform exploratory factor analysis (EFA) and confirmatory factor analysis (CFA), separately. A factor analysis was performed on the correlation matrix (bootstrap estimation) of the AIS item scores. The principal axis factoring method of factor extraction, along with Promax rotation (Kaiser normalization), was used in EFA. According to standard practices, multiple methods of factor retention, namely, the cumulative variance explained rule (>40%), Kaiser’s criteria (eigenvalue ≥1), Scree test and the robust parallel analysis based on minimum rank were used [20]. A parallel analysis with minimum rank was performed using Factor 10.8.04, and MS Excel was used to generate the graph from the tabular outcome. 

Comparative CFA was performed on models that have been reported by previous validation studies of AIS and the findings of EFA in this study. In brief, comparative CFA was performed on these models; model-A: 1-factor (Soldatos et al. [16]; Gomex-Benito et al. [21]), model-B: 2-factor model (Chung et al. [22]; Yen et al. [23]), model-C: 2-factor (Okajima et al. [24]) and model-D: 3-factor model (EFA finding in this study). According to recommended norms, fit indices from multiple categories were used [20,25]. Three absolute fit indices, i.e., Akaike’s information criterion (AIC), goodness-of-fit index (GFI), and χ^2^, χ^2^/df, were used. A non-centrality-based index, namely, the comparative fit index (CFI) was employed. Additionally, a relative fit index called Bollen’s incremental fit index (IFI) was also used [20,25]. For χ^2^/df, a value equal or less than 3 indicated an acceptable fit [26]. For, each of these, i.e., CFI, IFI, and GFI a value equal to or above 0.95 indicated excellent fit, while a value equal to or above 0.90 indicated acceptable fit [20,26]. A non-significant χ^2^-test was taken as suggestive of absolute fit [20,26]. A lower value of AIC implies a relatively better fit for the model [20,26]. A multi-group CFA was used to determine measurement invariance (configural, metric and scalar) of the validated model across gender groups. Chronbach’s alpha was used to assess internal consistency.

## 4. Results

### 4.1. Participants’ Characteristics 

Table 1 describes the characteristics of the study population of occupational computer users. The 424 study participants consisted of 202 (47.6%) males and 222 (52.4%) females. Most of the participants (68.6%; *n* = 291) were in the 20 to 29 years age group. Regarding BMI, a majority of the participants (63.7%) were in the normal weight category. Concerning marital status, educational qualification and smoking habit, most of the participants were unmarried (60.1%; *n* = 255), undergraduates (48.1%; *n* = 204) and nonsmokers (94.8%; *n* = 402), respectively. The participants’ mean AIS total score was 2.61 ± 2.18.

### 4.2. Factorial Validity

#### 4.2.1. Measures Showing Sample Suitability for Factor Analysis

The AIS scores in both the sub-samples, i.e., the EFA-sub-sample and CFA-sub-sample of the occupational computer users, met the requirements for factor analysis. There were sufficient linear combinations between the AIS item scores, as shown by the results of the Bartlett sphericity test (<0.001) [27]. The problems of multicollinearity and singularity were absent in the AIS item scores, as suggested by the determinant score (EFA-sub-sample: 0.21 and CFA-sub-sample: 0.26) [27]. The Kaiser–Meyer–Olkin test of sampling adequacy (EFA-sub-sample: 0.68 and CFA-sub-sample: 0.65) demonstrated that a mediocre level of shared variance existed among the AIS item scores [27]. All the item scores displayed adequate communality conditions, i.e., all were above 0.2 in both sub-samples (Table 2) [28]. Furthermore, all the diagonal elements in the anti-image matrix in both the sub-samples were above 0.5 [27]. 

#### 4.2.2. Exploratory Factor Analysis

**Figure 1:**
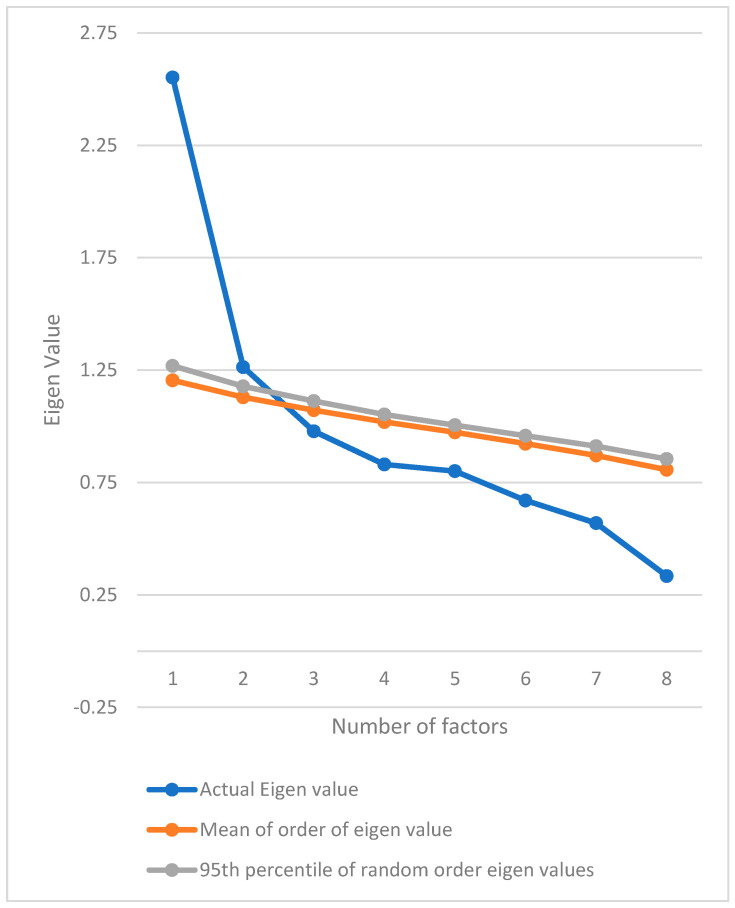
Parallel analysis sequence plot of the Athens Insomnia Scale (AIS) scores among occupational computer users in India.

The findings of EFA are presented in Table 3 and Figure 1. The results of the four-factor retention measures were non-unanimous; cumulative variance rule (>40%) and parallel analysis (minimum rank) found 2-Factor structures, while Kaiser’s rule and scree test revealed a 3-factor model (Table 3, Figure 1). 

The factor loadings of the AIS items ranged from 0.26 to 0.99 (Table 4). According to the 3-factor model (as indicated by the Kaiser’s rule and Scree test); the items assessing sleep induction, total sleep duration and sleep quality loaded on factor-1, daytime impact due of sleep disturbances (wellbeing, functioning capacity and daytime sleepiness), loaded on factor-2 and items assessing night time symptoms (awakenings during the night and early awakenings) loaded on factor-3 (Table 4).

#### 4.2.3. Confirmatory Factor Analysis

**Figure 2:**
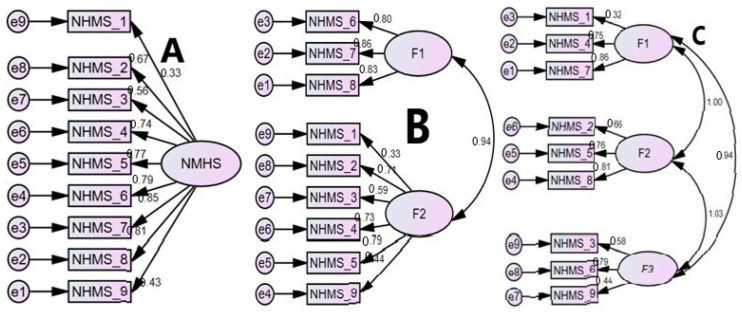
Confirmatory factor analysis models of the Athens Insomnia Scale (AIS) scores in Indian computer professionals. A: 1-Factor (Soltatos et al.; Gomex-Benito et al.), B: 2-Factor model (Chung et al.; Yen et al.), C: 2-Factor (Okajima et al.) and D: 3-Factor model (EFA finding in this study). All coefficients are standardized. *Ovals* latent variables, *rectangles* measured variables, *circles* error terms, *single-headed arrows* between *ovals* and *rectangles* factor loadings, *single-headed arrows* between *circles* and *rectangles* error terms.

The 3-factor model (model-D; Figure 2) was supported by most of the fit statistics; this model had excellent fit according to GFI (0.95), acceptable fit according to IFI and nearly acceptable fit, as determined by CFI (0.89) (Table 5). The value of the relative fit index of AIC was also least for the 3-factor model (model-D), additionally, the χ^2^/df demonstrated an ideal range (2.61) (Table 5) [20,25,26]. 

#### 4.2.4. Measurement Invariance

Model-D: Measurement invariance among gender groups

The 3-factor model supported by CFA, i.e., model-D was found to have a configural invariance across gender groups: CFI was above 0.96, RMSEA less than 0.5 and χ^2^/df < 3, when the model was compared across gender without limitations (Table 6). Results supporting metric invariance were found: a non-significant χ^2^ test of difference (*p* = 0.197), and ΔCFI < 0.01, when fully unconstrained model-D was compared to a model-D with equal loadings (Table 6). The results supported scalar invariance: a non-significant χ^2^ test of difference (*p* = 0.248), and ΔCFI < 0.01, when fully unconstrained model-D was compared to a model-D with equal intercepts (Table 6). Strict invariance was not supported for model-D; significant χ^2^ test of difference (*p* < 0.001), the absolute value of ΔCFI > 0.01, when fully unconstrained model-D was compared to a model-D with equal factor invariance (Table 6)

### 4.3. Preliminary Item Analysis and Internal Consistency 

The descriptive statistics, internal homogeneity, and item discrimination are presented in Table 7. There were no missing values in AIS item scores in the study sample. All the AIS item scores demonstrated a floor effect; the lowest score occurred in more than 15% of the sample. None of the AIS item scores showed a ceiling effect; the highest score occurred in less than 15% of the sample. The univariate distribution demonstrated skewness for all items (skewness > 1), except sleep induction and daytime sleepiness, and kurtosis for items (kurtosis < 1), other than sleep induction, final awakening earlier and daytime sleepiness. The internal homogeneity and item discrimination were determined by item-total correlations (0.37–0.62), corrected item-total correlations (0.23–0.53) and Cronbach’s alpha if item deleted (0.57-.66) (Table 6). The internal consistency of the AIS in this sample of occupational computer users was 0.66. 

## 5. Discussion

This is the first study to determine the psychometric properties of AIS (8-item—original version) in Indian computer professionals who are at increased risk of insomnia. The study evaluated the AIS’s factorial validity, internal consistency and item discrimination in an earlier uninvestigated population—Indians. This study showed that when administered to occupational computer users in India; the three-dimensional model of the AIS demonstrated sufficient factorial validity, adequate internal consistency, strong internal homogeneity, and adequate item discrimination. In general, few studies have explored the factorial validity of sleep-evaluating questionnaires among Indians [25,29,30]. 

### 5.1. Measures Showing Sample Suitability for Factor Analysis

All the five measures used in the present study, i.e., communality criteria, the majority of inter-item correlations that were above 0.3, KMO, Bartlett’s test, determinant, and diagonal elements of the anti-image matrix unanimously confirmed the suitability of the application of factorial validity procedures in the AIS item score in this sample of occupational computer users [20]. Previous studies by Soldatos et al., Gomez-Benito et al., Chung et al., Yen et al., and Okajima et al. investigated the factorial validity of AIS (original or translated versions) [16,21,22,23,24]. However, none of these studies reported on any of these measures, which would have confirmed whether the data in their studies were suitable for performing a factor analysis. Few previous studies have performed both EFA and CFA to determine the dimensionality of the AIS. Of the studies cited above, only Yen et al. used two separate sub-samples for EFA and CFA [23]. 

### 5.2. Exploratory Factor Analysis

The findings from EFA were heterogeneous; inasmuch as both a 2-factor and a 3-factor structure were supported by two measures of factor retention. The 3-factor structure of the AIS found in this study is novel and indicates three underlying dimensions of the insomnia symptoms, namely, nighttime symptoms (Sleep induction, total sleep duration, and sleep quality), awakening issues (awakenings during the night, early awakening) and daytime impact, due to sleep disturbances (wellbeing, functioning capacity and daytime sleepiness). This three-dimensional finding is slightly different from the 2-dimensional nature of the insomnia proposed by Soldatos et al. [16,17] Okajima et al. also identified a two-dimensional model of AIS among a Japanese population [24]. The studies by Soldatos et al., Gomez-Benito et al., Chung et al., and Okajima et al. did not employ a parallel analysis for the retention of factors in EFA [20,21,22,24]. This is contrary to recommended norms regarding the use of factor retention methods during EFA [20,25]. 

### 5.3. Confirmatory Factor Analysis

Fit indexes further validated the 3-factor structure (model-D) of the AIS in this study sample. Therefore, in all, two measures of factor retention, i.e., Kaiser’s criteria and Scree test, along with fit indices which favored the validity of model-D, a 3-factor structure. Most of the previous studies investigating the dimensionality of AIS did not report fit indexes from multiple categories [16,24]. This is contrary to recommended norms regarding the use of fit indexes, i.e., multiple fit indexes from different categories should be employed. This further complicates a comparison of results from the previous study with that of the current study [20,25]. 

### 5.4. Measurement Invariance

To further establish the validity of the 3-factor model, measurement invariance across gender groups was determined. Measurement invariance supported configural, metric and scalar invariance of the 3-factor model in the study population. No previous study has evaluated measurement invariance of a factor structure of the original English version of the AIS (8-items), however, recently, Iwasa et al. reported multigroup-CFA findings of the Japanese version of the AIS [31]. A direct comparison is not possible, because of: (i) differences in the AIS version used, (ii) methodological differences, i.e., Iwasa et al. did not perform EFA, and (iii) differences in the dimensionality of AIS validated in both studies. Future studies using multi-centric data which take into account the methodological discrepancies of some of the previous studies are needed. 

### 5.5. Internal Consistency and Item Discrimination

Internal consistency, as indicated by Cronbach’s alpha, was satisfactory. The Cronbach’s alpha reported in the present study was lower than those reported by Chung et al. (0.81), Soldatos et al. (0.89) and Gomez-Benito et al. (0.86) [16,21,22]. 

### 5.6. Preliminary Item Analysis 

The floor effect for all the AIS scores is reflective of the normative sleep behavior in the majority of the study population. This finding is understandable because the study subjects were drawn from a non-clinical population. Similar generalizations about floor/ceiling effects in the normal populations have been made for questionnaires measuring other clinical features [20]. This finding about values of all the Cronbach’s alpha if items were deleted, item-total correlations and corrected item-total correlations being greater than 0.2; supports the conclusion that the AIS score was based on adequate item discrimination in the study population. This implies that AIS item scores can differentiate between low and high scorers, even at both the extremes [20]. Soldatos et al. reported item-total correlations in the range of 0.53 to 0.75 among psychiatric patients and normal participants in Greece [16]. In a study of students, psychiatric patients and community members in Spain, Gomez-Benito et al. reported a similar range of correlations, i.e., 0.39 to 0.86 [21]. In a study of Chinese adolescents, Chung et al. reported correlations ranging from 0.46 to 0.78 [22]. The findings of these studies were consistent with those of the present study in supporting adequate item discrimination of the AIS in different populations. 

## 6. Limitations and Recommendations

The limitations of this study were that measures of construct validity and diagnostic validity were not employed. Item analysis was done based on Classical theory methods. Future studies based on RASCH analysis are recommended. 

## 7. Conclusions

The AIS is composed of a three-factor structure that supports a three-dimensional perspective of insomnia among occupational computer users. The findings of the present research support the validity of AIS to screen insomnia reflected by psychometric properties, such as internal consistency, internal homogeneity, item discrimination, and factorial validity.

## Figures and Tables

**Table 1:** Participant Characteristics.

Characteristics	Mean ± SD/Frequency (%)
Age (Years)	
20–29	291 (68.6)
30–39	91 (21.4)
40 and above	42 (10)
Gender	
Male	202 (47.6)
Female	222 (52.4)
Body Mass Index	
Less than 18.5 (Under Weight)	85 (20)
18.5–24.99 (Normal)	270 (63.7)
25–29.99 (Over Weight)	61 (14.4)
30 or Above (Obesity)	8 (1.9)
Marital Status	
Unmarried	255 (60.1)
Married	167 (39.4)
Widow	2 (0.5)
Education Level	
Higher secondary/Pre-university College	54 (12.7)
Diploma	48 (11.3)
Under Graduate	204 (48.1)
Post Graduate	109 (25.7)
Others	9 (2.1)
Smoking	
Yes	22 (5.2)
No	402 (94.8)
Total AIS	2.61 ± 2.18

SD: Standard Deviation, AIS—Athens Insomnia Scale.

**Table 2:** Sample size adequacy measures of the Athens Insomnia Scale (AIS) scores in Indian computer professionals.

Measures	Values		
	Total Sample	CFA Sub-Sample	EFA Sub-Sample
Anti-image matrix	0.63–0.79	0.58–0.73	0.64–0.86
Bartlett’s test of Sphericity	Χ^2^(28) = 555.0, *p* < 0.001	Χ^2^(28) = 277.20, *p* < 0.001	Χ^2^(28) = 327.00, *p* < 0.001
Determinant	0.27	0.26	0.21
Kaiser-Meyer-Olkin Test of Sampling Adequacy (KMO)	0.70	0.65	0.68
Communality *	0.37–0.58	0.38–0.74	0.39–0.74

CFA confirmatory factor analysis. * Principal Component Analysis for the unrotated solution.

**Table 3:** Summary of the factor extraction measures used in exploratory factor analysis of the Athens Insomnia Scale (AIS) scores in Indian computer professionals.

Number of Factors	Eigenvalue	Cumulative Variance Explained (%)	Above Point of Inflection on Scree Plot	Decision to Extract		
				Kaiser’s Criteria (Eigenvalue ≥ 1)	Cumulative Variance Rule (>40%)	Scree Test
1	2.69	33.65	Yes	√	√	√
2	1.17	48.28	Yes	√	√	√
3	1.01	60.90	Yes	√	Χ	√
4	0.83	71.30	No	Χ	Χ	Χ

√ indicates extraction criteria fulfilled, Χ indicates otherwise.

**Table 4:** Pattern matrix of the Athens Insomnia Scale (AIS) scores in Indian computer professionals.

Items of the AIS	Factor-1	Factor-2	Factor-3
Sleep Induction	0.26		
Awakenings During the Night			0.88
Final_awake			0.33
sleep_dur	0.99		
sleep_qual	0.64		
wellbeing_day		0.43	
Funtion_day		0.82	
sleepiness_day		0.29	

Principal Axis Factoring extraction with Promax rotation (Kaiser Normalization). Factor loading less than 0.25 was removed from interpretation.

**Table 5:** Fit statistics of the Athens Insomnia Scale (AIS) scores in Indian computer professionals.

Models	IFI	CFI	GFI	AIC	χ^2^	df	*p*	χ^2^/df
A	0.73	0.72	0.90	123.74	91.74	20	<0.01	4.59
B	0.80	0.79	0.92	106.84	72.84	19	<0.01	3.83
C	0.85	0.84	0.93	92.91	58.91	19	<0.01	3.10
D	0.90	0.89	0.95	82.40	44.43	17	<0.01	2.61

A: 1-Factor (Soltatos et al.; Gomex-Benito et al.), B: 2-Factor model (Chung et al.; Yen et al.), C: 2-Factor (Okajima et al.) and D: 3-Factor model (EFA finding in this study). CFI: Comparative Fit Index, GFI: Goodness of fit index, AIC: Akaike information criterion, IFI: Bollen’s Incremental Fit Index.

**Table 6:** Measurement invariance of the 3-factor model of the Athens Insomnia Scale (AIS) among occupational computer users in India.

	*Χ* ^2^	df	*p*-Value	*Χ*^2^/df	CFI	RMSEA	*Χ*^2^ Difference Test Statistics			ΔCFI
							Δ*Χ*^2^	Δdf	*p*-Value	
Equal form/Configural invariance	55.740	34	0.011	1.639	0.960	0.039				
Metric invariance-Equal loadings	63.077	39	0.009	1.617	0.956	0.038	7.338	5	0.197	−0.004
Scalar invariance-Equal intercepts	73.330	47	0.008	1.560	0.956	0.036	10.253	8	0.248	0.000
Strict invariance-Equal factor variances	163.953	61	0.000	2.688	0.811	0.063	90.623	14	0.000	−0.145

**Table 7:** Descriptive statistics, communality, item-discrimination, factor loadings of the Athens Insomnia Scale (AIS) among occupational computer users in India.

Items of the AIS	Skewness	Kurtosis	Cronbach’s Alpha If Item Deleted	Item-Total Correlation	Corrected Item-Total Correlation	Percentage Distribution Across Item Scores			
						0	1	2	3
Sleep induction	0.62	0.57	0.66	0.56 **	0.26	40.8	52.6	5.7	0.9
Awakenings during night	1.34	1.60	0.59	0.59 **	0.47	65.4	30.7	3.3	0.5
Final awakening earlier	1.17	0.88	0.66	0.50 **	0.22	64.9	32.1	2.6	0.2
Total sleep duration	1.71	3.15	0.57	0.62 **	0.53	72.4	25.2	1.9	0.5
Sleep quality	2.30	5.95	0.58	0.59 **	0.52	79.7	17.9	1.9	0.5
Daytime wellbeing	2.79	5.79	0.64	0.37 **	0.34	79.5	17.9	1.9	0.5
Daytime functioning capacity	4.09	19.28	0.63	0.38 **	0.33	79.5	17.9	1.9	0.5
Daytime sleepiness	0.93	0.85	0.66	0.44 **	0.23	59.0	39.4	1.2	0.5

D: Standard deviation; SE: Standard Error. AIS: Athens Insomnia Scale. ** *p* < 0.01. Cronbach’s alpha for the AIS: 0.66.
